# A Novel QTL for Resistance to Phytophthora Crown Rot in Squash

**DOI:** 10.3390/plants10102115

**Published:** 2021-10-06

**Authors:** Vincent Njung’e Michael, Yuqing Fu, Swati Shrestha, Geoffrey Meru

**Affiliations:** The Tropical Research and Education Center, Horticultural Sciences Department, University of Florida, 18905 SW 280 ST Homestead, Gainesville, FL 33031, USA; michael.vn@ufl.edu (V.N.M.); yuqingf@ufl.edu (Y.F.); s.shrestha@ufl.edu (S.S.)

**Keywords:** QTL mapping, *Cucurbita pepo*, breeding, disease resistance, *Phytophthora capsici*, marker-assisted selection

## Abstract

*Phytophthora capsici* Leonian causes significant yield losses in commercial squash (*Cucurbita pepo*) production worldwide. The deployment of resistant cultivars can complement integrated management practices for *P. capsici*, but resistant cultivars are currently unavailable for growers. Moderate resistance to Phytophthora crown rot in a selection of accession PI 181761 (*C. pepo*) (designated line #181761-36P) is controlled by three dominant genes (R4, R5 and R6). Introgression of these loci into elite germplasm through marker-assisted selection (MAS) can accelerate the release of new *C. pepo* cultivars resistant to crown rot, but these tools are currently unavailable. Here we describe the identification of a quantitative trait locus (QTL), molecular markers and candidate genes associated with crown rot resistance in #181761-36P. Five hundred and twenty-three SNP markers were genotyped in an F_2_ (*n* = 83) population derived from a cross between #181761-36P (R) and Table Queen (S) using targeted genotyping by sequencing. A linkage map (2068.96 cM) consisting of twenty-one linkage groups and an average density of 8.1 markers/cM was developed for the F_2_ population. The F_2_:_3_ families were phenotyped in the greenhouse with a virulent strain of *P. capsica*, using the spore-spray method. A single QTL (*QtlPC-C13*) was consistently detected on LG 13 (chromosome 13) across three experiments and explained 17.92–21.47% of phenotypic variation observed in the population. Nine candidate disease resistance gene homologs were found within the confidence interval of *QtlPC-C13*. Single nucleotide polymorphism (SNP) markers within these genes were converted into Kompetitive Allele Specific PCR (KASP) assays and tested for association with resistance in the F_2_ population. One SNP marker (C002686) was significantly associated with resistance to crown rot in the F_2_ population (*p* < 0.05). This marker is a potential target for MAS for crown rot resistance in *C. pepo*.

## 1. Introduction

*Phytophthora capsici* is a soil-borne oomycete pathogen with a wide host range, including all economically important species of *Cucurbita* (*C. pepo* L., *C. moschata* Duchesne, and *C. maxima* Duchesne) [1]. It is responsible for foliar blight, fruit rot, root rot and crown rot disease syndromes in squash, and is most severe in the commercial production of *C. pepo* [2]. Phytophthora crown rot is prevalent in production areas that experience frequent flooding, which facilitate proliferation of motile infectious *Phytophthora* zoospores that result in damping off and severe damage in young seedlings and older plants, respectively [3,4]. 

Effective chemical management of *P. capsici* is hindered by the evolution of pathogen populations insensitive to approved fungicides [5,6]. Moreover, cultural control through crop rotation or management of standing water in the field is inadequate because *P. capsici* endures in the soil as persistent oospores [7,8]. Availability of host resistance could complement integrated pest management strategies for the pathogen; however, no resistant commercial *C. pepo* cultivars are currently available [9,10]. Commercial cultivars of *C. pepo* can be categorized into eight edible cultivar-groups belonging to either subspecies pepo (Zucchini, Pumpkin, Vegetable Marrow, Cocozelle) or subspecies ovifera (Straightneck, Acorn, Crookneck, Scallop) [11,12]. These cultivar groups exhibit considerable phenotypic differences, both in horticultural traits and susceptibility to Phytophthora crown rot. A comparative screening of commercially sourced cultivars representing the two subspecies (10 ssp. *ovifera* and 12 subsp. *pepo*) revealed higher resistance in *C. pepo* subsp. *pepo* (mean DS = 3.11) than *C. pepo* subsp. *ovifera* (mean DS = 4.92) [13]. Padley et al. [14] identified 16 *C. pepo* accessions with moderate to high resistance against Phytophthora crown rot, among which PIs 181761 and 615132 were the most resistant. Further selection and selfing of PI 181761 yielded a breeding line (designated #181761-36P) fixed for resistance against Phytophthora crown rot. Resistance in #181761-36P is controlled by three dominant genes designated R4, R5 and R6 [15]. Marker-assisted selection (MAS) for Phytophthora crown rot resistance in #181761-36P would greatly expedite breeding and the release of resistant commercial cultivars. However, the genomic loci, molecular markers and candidate genes associated with Phytophthora crown rot resistance in #181761-36P are currently unknown. 

Advances in next generation sequencing technologies has facilitated the development of genomic tools for genetic studies in *C. pepo*, including a transcriptome assembly [16], genetic linkage maps [17,18], and recently, a reference genome [19]. These tools, coupled with a relatively small diploid genome (approximately 263 Mb), provide an opportunity for the discovery of marker-trait associations in *C. pepo*. QTL mapping enables identification of genomic regions contributing to phenotypic variation in segregating populations [20,21] by anchoring phenotypes to sections of a genetic map, generated using polymorphic markers in linkage disequilibrium [22,23]. In *C. pepo*, QTL mapping has been extensively used to identify genomic regions associated with economically important traits. For example, an interspecific linkage map constructed with random amplified polymorphic DNA (RAPD) markers from a backcross population of yellow Straightneck inbred A0449 (*C. pepo*) and Nigerian Local (*C. moschata*) revealed QTLs associated with various qualitative (silver mottling, precocious yellow fruit and rind color intensity) and quantitative (fruit shape and depth of indentations) traits [24]. Later, a *C. pepo* genetic map comprising RAPD, amplified fragment length polymorphism (AFLP), simple-sequence repeats (SSR) and sequence-characterized amplified region (SCAR) markers was used to identify QTLs for mottled leaves, hull-less seeds and bush growth habit in two F_2_ populations [25]. To improve the genome coverage of the two genetic maps, Gong et al. (2008) added more SSR markers to generate a linkage map consisting of 21 linkage groups. The first SNP-based *C. pepo* genetic map utilized an Illumina GoldenGate 384-SNP assay to detect 48 QTLs associated with 42 traits in an F_2_ population from a cross between Zucchini (subsp. *pepo*) × Scallop (subsp. *ovifera*) cultivar groups [18]. Analysis of recombinant inbred lines advanced from the same cross with 7718 SNPs yielded a high-density linkage map that allowed identification of 48 QTLs across 43 traits [26]. Recently, Vogel et al. [10] combined linkage mapping with BSA-Seq to identify six QTLs associated with resistance to Phytophthora root and crown rot in *C. pepo* in an F_2_ population derived from a cross between #Pc-NY21 (resistance derived from PI 615089) and #Dunja F_1_ (a susceptible zucchini cultivar). Two of these QTLs were syntenic to genomic regions associated with crown rot resistance in *C. moschata* [27], suggesting a common evolutionary origin of resistance in *Cucurbita* [10]. 

The goal of the current study was to identify QTL, DNA markers and candidate genes associated with Phytophthora crown rot resistance in #181761-36P. These tools will not only allow efficient introgression of #181761-36P resistance into elite cultivars through MAS, but also facilitate pyramiding with other resistance QTLs previously identified in *C. pepo* [10].

## 2. Materials and Methods

### 2.1. Plant Material, DNA Extraction and SNP Genotyping

An intersubspecific cross between #181761-36P (resistant; *C. pepo* subsp. *pepo*) and Table Queen (susceptible; *C. pepo* subsp. *ovifera*) was made in the greenhouse and a single F_1_ plant was selfed to yield F_2_ plants, which were further selfed to generate 83 F_2:3_ families. Extraction of DNA from the leaf material of the parents, the F_1_ and each of the F_2_ plants was achieved using the E.Z.N.A kit (Omega Biotek, Norcross, GA, USA), according to the manufacturer’s instructions. The concentration and quality of the DNA was determined by absorbance measurements (NanoDrop 8000; Thermo Fisher Scientific, Waltham, MA, USA) and agarose gel (0.8% *w/v*) electrophoresis. 

Six hundred and five publicly available SNP markers [16,18] were selected for genotyping (Table S1). These SNPs were within genic regions and evenly distributed across the genome. Among these, 83 SNP markers were unsuitable for probe design, thus only 523 markers were genotyped in the parents, F_1_ and F_2_ individuals using the targeted genotyping by sequencing platform (SeqSNP technology; LGC Genomics, Hoddesdon, UK). Briefly, the *C. pepo* reference genome [19] was used to develop a library of oligo probes (average 60 bp) flanking each SNP of interest. Sequencing libraries (1 × 75 bp) were prepared and run on a NextSeq 500 Illumina Next Generation Sequencing platform. Sequence reads were mapped onto the *C. pepo* reference genome and SNP calling was performed using standard bioinformatic tools [28,29,30].

### 2.2. Inoculation and Phenotyping

*P. capsici* inoculum was prepared from a virulent isolate #121 (provided by Dr. Pamela Roberts, University of Florida, Gainesville, FL, USA) according to the method described by Krasnow et al. [13], with slight modifications. Briefly, 5-mm cornmeal mycelial agar plugs of *P. capsici* were transferred to 14% V8 agar plates (140 mL V8 juice, 3 g CaCO3, 16 g agar per liter) and cultured under constant fluorescent light at 28 °C. On the 7th day, the plates were flooded with cold sterile distilled water (4 °C), and chilled at 4 °C for 30 min prior to incubation at 21 °C for 60 min to allow release of zoospores synchronously. Zoospores in the inoculum suspension were quantified with a hemocytometer and adjusted to 2.0 × 10^4^ zoospores/mL. 

Twelve seeds, each of the F_2:3_ families (*n* = 83); 40 seeds of each parent and 10 seeds each of the F_1_ individuals, were sown in the greenhouse in 4-inch diameter pots filled with sterilized Proline C/B growing mix (Jolly Gardener, Quakertown, PA, USA) amended with a slow-release fertilizer (14N-4.2P-11.6K) (Osmocote; Scotts, Marysville, OH, USA). Twelve seeds of the resistant *C. moschata* breeding line #394-1-27-12 [31] were also included in each experiment as checks. The experiment was arranged in an incomplete block design with 10 seeds of both parents included as controls in each block. At the third true-leaf stage, a hand spray bottle adjusted to release 0.5 mL volume per spray was used to deliver 1.5 mL of zoospore suspension at the crown of each plant. Visual recording of disease severity was done every three days from six days post inoculation (dpi) to 28 dpi using a scale of 0 to 5 whereby a rating of 0 was assigned to plants with no symptoms, 1 for plants with a small brown lesion at the base of the stem, 2 for plants with a lesion progressed up to the cotyledons causing constriction at the base, 3 for partially collapsed plants with apparent wilting of leaves, 4 for completely collapsed plants exhibiting severe wilting, and 5 for dead plants [14]. Plants having a score of 1 or less at 28 dpi were classified as resistant, whereas those having a score ≥2 were classified as susceptible [31]. Area Under Disease Progress Curve (AUDPC) values for the F_2:3_ families were determined using the trapezoidal integration method [32] and used for QTL mapping. The experiment was carried out thrice.

### 2.3. Linkage Map Construction and QTL Analysis

A genetic linkage map was constructed with Onemap package in R software (Vienna, Austria) with SNP markers polymorphic between the parents [33,34]. SNP markers with significant segregation distortion from the expected Mendelian segregation (1:2:1) as determined through χ^2^ test were excluded. Linkage groups were constructed using the Kosambi mapping function by exploiting recombination fractions [35]. This was done by choosing three initial markers using rapid chain delineation and sequentially adding markers that map with a significant LOD threshold of three [36]. Alternative marker orders were considered with the same LOD threshold before assembling the final linkage map. QTL mapping was performed by Haley–Knott linear regression of AUDPC values against genotype probabilities calculated from the linkage map as implemented in the R/qtl2 package [37]. QTL analysis was conducted independently for each experiment, while joint analysis was conducted using the mean data across experiments. Likelihood-odds (LOD) thresholds set by 1000 permutations (α = 0.05) were used to determine the statistical significance of a QTL [38]. Additive and dominance effects, as well as the proportion of total phenotypic variance explained by the QTLs were also estimated. The QTL were visualized using MapChart software (Wageningen, The Netherlands) [39].

### 2.4. Marker Test and Candidate Gene Identification

Five SNP markers (Table S2) within the confidence interval of the detected QTL were converted into Kompetitive allele specific (KASP) PCR assays [40] and genotyped in the F_2_ population. KASP oligonucleotides were designed using BatchPrimer3 software (Albany, CA, USA) [41], and the PCR assays were performed in 10-μL reactions containing 5-μL of 2× low ROX KASP master mix (LGC Genomics LLC., Teddington, UK), 0.16 μL each of forward primers (10 μM), 0.41 μL of reverse primer, 2 μL of genomic DNA (50 ng/μL) and 2.27 μL of H_2_O. The PCR conditions consisted of an initial incubation at 94 °C for 15 min, a touchdown PCR at 94 °C for 20 s, 61 °C for 60 s, with a 0.6 °C decrease per cycle for 10 cycles, followed by 26 cycles of 94 °C for 20 s and 55 °C for 60 s. Fluorescent end-point readings and cluster calling were performed using LightCycler^®^ 480 Instrument II (Roche Life Sciences, Penzberg, Upper Bavaria, Germany). Marker-trait associations were tested using the Kruskal-Wallis test (*p* ≤ 0.05) in R statistical software [34]. Candidate genes within the significant QTL interval were identified by scanning the corresponding genomic region for disease resistant homologs using the *C. pepo* reference genome [19].

## 3. Results

### 3.1. Phenotypic Analysis

**Figure 1:**
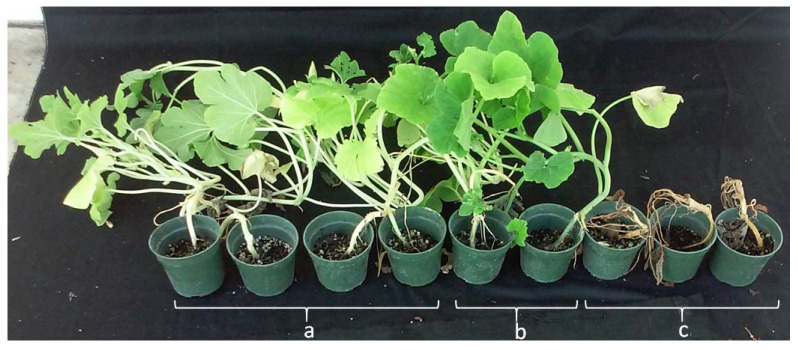
Resistance to Phytophthora crown rot in breeding line (**a**) #181761-36P and (**b**) #394-1-27-12, and susceptibility in (**c**) Table Queen cultivar at 28 days post inoculation.

At 28 dpi, #181761-36P plants exhibited high resistance to Phytophthora crown rot (mean DS = 0.55), whereas the susceptible parent (Table Queen) rapidly succumbed to the pathogen (mean DS = 5) (Figure 1). The resistant breeding line #394-1-27-12 (*C. moschata*) remained asymptomatic throughout the experiment (mean DS = 0) (Figure 1).

**Figure 2:**
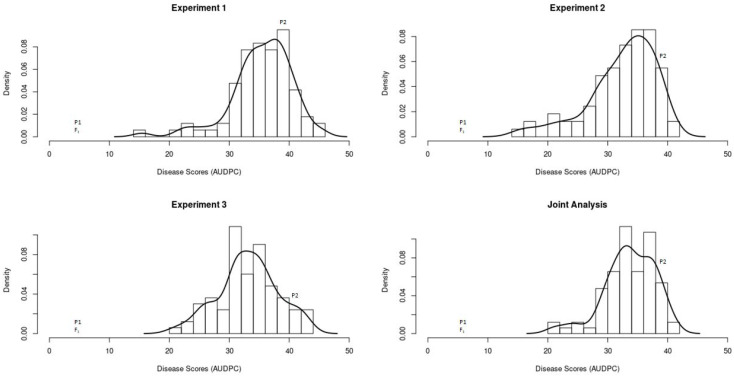
Frequency distribution for disease severity [area under disease progress curve (AUDPC)] in the F_2:3_ population for experiment 1, experiment 2, experiment 3 and joint analysis (mean across 3 experiments). The resistant parent (#181761-36P), susceptible parent (Table Queen) and F_1_ are indicated by P1, P2 and F_1,_ respectively.

The mean AUDPC values for the F_2:3_ families across the three experiments ranged from 21.18 to 40.69 and displayed a slightly left-skewed normal distribution (Pearson coefficient of skewness = −0.7563) (Figure 2). Transgressive segregation was observed in one direction, with some F_2:3_ families showing higher susceptibility than the susceptible parent (Figure 2). Significant positive correlations (*p* < 0.05) were observed for AUDPC values among the three experiments and ranged between 0.57 to 0.65. 

### 3.2. SNP Analysis and Map Construction

Targeted genotyping by sequencing yielded 24,933,788 reads averaging approximately 129,858 reads per sample, effectively giving a 231× coverage for each target SNP. SNP markers that were heterozygous (*n* = 68) in the parents, monomorphic (*n* = 182) between the parents or those that deviated (*p* < 0.00001) from the expected segregation ratio of 1:2:1 (*n* = 29) was excluded from linkage mapping. The complete genetic map comprised 21 linkage groups encompassing 2068.96cM with a marker density of 8.1 SNP/cM (Table 1 and Figure S1). The linkage map covered approximately 81.1% of the total *C. pepo* reference genome [19], while coverage of individual chromosomes ranged from 52.1% to 99.4% for chromosomes 16 and 1, respectively (Table S3); excluding chromosome 17 that only had two markers.

### 3.3. QTL Detection, Candidate Genes and Marker Validation

**Figure 3:**
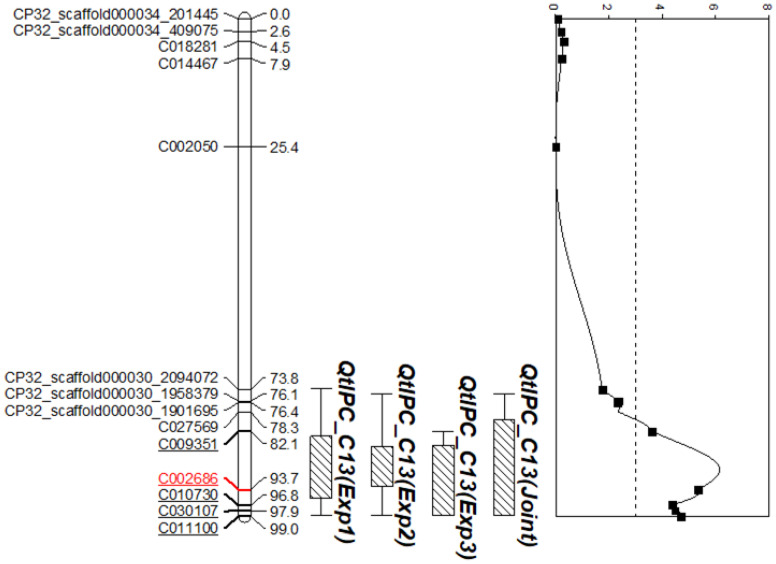
Quantitative trait loci (QTL) associated with resistance to Phytophthora crown rot on chromosome (LG) 13 in the #181761-36P × Table Queen F_2:3_ population. The number in parenthesis after the QTL name indicates the experiment in which it was mapped, while ‘Joint’ represents locus detected using the mean AUDPC across the three experiments. The phenotypic variation explained by the QTL ranged from 17.9% to 21.5% in the F_2:3_ families. The markers and their corresponding positions (cM) are to the left and right of the chromosome, respectively. Underlined markers are those within the QTL interval, while the significant marker (C002686) is indicated in red font. The QTL peak represented on the far right was drawn using data from joint analysis. The LOD threshold is indicated by a dotted line. The figure was generated using MapChart, version 2.2 (Wageningen, Netherlands) [39].

Analyses with data from the three experiments, and from joint analysis, consistently detected a significant QTL (*QtlPC-C13*) on chromosome 13 (Table 2 and Figure 3). This QTL explained 17.9% to 21.5% of the phenotypic variation observed in F_2:3_ families, with likelihood-odds values ranging from 3.1 to 5.9 (Table 2). The peak SNP (C002686) for *QtlPC-C13* was consistent across the three experiments and the joint analysis. The interval for *QtlPC-C13* spanned between 1.07 Mb (Experiment 2) and 1.85 Mb (Joint Analysis) and contained five SNPs (LOD = 3.65 to 5.91) (Table S2), each within or near candidate disease defense-related genes. The QTL interval contained a total of 23 genes among which 9 were annotated as candidate disease resistance genes (Table S4). The peak SNP C002686 was located within the intron of *Cp4.1LG13g07770.1* gene that produces a quinone oxidoreductase-like protein-2 homolog. Downstream of SNP C002686 is *Cp4.1LG13g07410*, a gene encoding a Basic helix-loop-helix (BHLH) transcription factor, while *Cp4.1LG13g08020, Cp4.1LG13g08190* and *Cp4.1LG13g09000* are located upstream and encode a RING/U-box superfamily protein, Methyl esterase-11 and L-aspartate oxidase, respectively. SNP C009351 lies within a dirigent protein gene *Cp4.1LG13g09560*, while SNPs C010730 and C011100 are located within *Cp4.1LG13g07250* and *Cp4.1LG13g11450*.1 genes encoding a Eukaryotic translation initiation factor 3 subunit F and a Chloroplastic group IIA intron splicing facilitator CRS1, respectively. On the other hand, SNP 30107 lies within *Cp4.1LG13g10990*, a gene encoding E3 ubiquitin-protein ligase (SDIR1). 

All five SNPs were converted into KASP assays for validation in the F_2:3_ population. Among the five markers, SNP marker C002686 was significantly associated with resistance to Phytophthora crown rot in the F_2:3_ population (Kruskal–Wallis rank sum test, *p*-value = 0.0009528). 

## 4. Discussion

### 4.1. Phenotypic Analysis 

The high resistance to Phytophthora crown rot observed in #181761-36P confirms this accession as a good source for resistance breeding in *C. pepo* [14,15]. On the contrary, the Table Queen cultivar was highly susceptible as evidenced by a rapid expansion of water-soaked lesions to the cotyledons and subsequent wilting and death. The phenotypic distribution in the F_2:3_ population was normal, but skewed towards susceptibility, thus supporting a three-gene model previously described for resistance in #181761-36P [15]. A similar skewed distribution was observed in a *C. pepo* F_2:3_ population derived from a cross between a crown rot resistant breeding line #Pc-NY21 and a susceptible cultivar (#Dunja F_1_) [10], indicating a similar but independent inheritance pattern in both resistant sources. Correlation across the three experiments was moderate (0.57–0.65) but significant, supporting the reliability of the modified spray inoculation protocol [13]. Unfortunately, other Phytophthora crown rot QTL mapping studies in squash [10,27] used only single phenotypic screens, thus it’s difficult to compare the repeatability of the screens and their effect on QTL detection. Correlations between experiments may be improved by screening a larger number of individuals to increase the accuracy of disease severity means in the F_2:3_ families [42]. Transgressive segregation was observed towards susceptibility, whereby some of the F_2:3_ families were outside the range of the susceptible parent. This may be explained by antagonistic additive effects, whereby both parents contribute alleles in one direction or by other mechanisms [43,44]. 

### 4.2. Linkage Mapping and QTL Detection

Although the genetics of resistance to Phytophthora crown rot in #181761-36P has been previously described [15], the QTL associated with the resistance are currently unknown. In the current study, a linkage map was constructed using 244 SNP markers to aide detection of QTL linked to Phytophthora crown rot resistance in #181761-36P. The length (2068.96 cM) and marker density (8.1 SNP/cM) obtained in the current study is similar to those reported for *C. pepo* populations in recent linkage mapping studies. Esteras et al. [18] developed a linkage map (1740.8 cM) consisting of 315 markers (304 SNP and 11 SSR markers) with a density of 5.56 cM/marker in an intersubspecific F_2_ population derived from a cross between subsp. *pepo* and *ovifera*. Montero-Pau et al. [26] used an intersubspecific recombinant inbred line (RIL) population (subsp. *pepo* × *ovifera*) to construct a linkage map (2817.6 cM) using 7718 SNP markers at a density of 6.02 cM/marker. Xiang et al. [45] used an intrasubspecific (subsp. *pepo*) RIL population to develop a linkage map (2199.1 cM) consisting of 2292 markers with a density of 3.78 cM/marker. More recently, Vogel et al. [10] used an intrasubspecific (subsp. *pepo*) F_2_ population to develop a linkage map (2023.38 cM) consisting of 605 SNP markers and a density of 3.88 cM/marker. Taken together, these studies suggest sufficient genetic diversity within and across subspecies of *C. pepo* that allow adequate polymorphism for linkage mapping. 

A major QTL (*QtlPC-C13*) associated with Phytophthora crown rot in #181761-36P was detected on chromosome 13 and explained up to 21.5% of the phenotypic variation observed in F_2:3_ population. The detection of *QtlPC-C13* across the three experiments and joint analysis indicates the consistency of the disease rating scale [14] and the modified spray inoculation protocol [13]. The phenotypic variation explained by *QtlPC-C13* is consistent with that of an oligogenic trait and supports previous genetic studies with Phytophthora in *Cucurbita*. Recently, Vogel et al. [10] identified six QTLs of minor to moderate effect (R^2^ values between 2–10%) associated with Phytophthora crown and root rot in *C. pepo* breeding line Pc-NY21 (resistance derived from PI 615089). However, the QTL identified in #181761-36P (*QtlPC-C13*) is novel and did not colocalize with any of the six QTLs in Pc-NY21 which were detected on chromosome 4, 5, 8, 12, 16 and 19 [10]. Three dominant genes designated R4, R5, and R6 control resistance in #181761-36P, with the R4 gene conferring resistance independent of the other two [15]. A similar three-gene (R1, R2 and R3) resistance model was proposed for Phytophthora crown rot resistance in *C. moschata* breeding line #394-1-27-12 [31], and the corresponding genomic loci were mapped on chromosome 4, 11 and 14 [27]. 

Although three genes are proposed for the resistance in #181761-36P, only one QTL was identified in the current study. This is perhaps due to the relatively small F_2_ population size (*n*= 83) used for QTL detection, which resulted from an unexpected segregation of male sterility in the F_2_ population that reduced the number of F_2:3_ families. Population size is an important factor in the ability to detect QTL, especially those of minor effects as previously reported in barley [46] and watermelon [47]. Furthermore, the linkage map developed in the current study covered 81.1% of the *C. pepo* genome, thus additional resistance loci may lie within missing chromosomal regions. For example, only 1.3% of LG (chromosome) 17 was represented in the linkage map due to a lack of informative transcriptome-based markers within the region (Table S3). To increase genome coverage and the resolution of the identified QTL, more markers should be anchored unto the missing chromosomal segments using recently available SNP markers [10,26] or novel markers derived from whole-genome resequencing in combination with QTL-seq [27,48]. 

### 4.3. Marker Validation and Candidate Genes 

Among the five SNP markers found within *QtlPC-C13*, C002686 was consistently detected as the peak SNP across all the mapping experiments, and was the only marker significantly associated with resistance to Phytophthora crown rot in the F_2:3_ population. C002686 lies within *Cp4.1LG13g07770.1* gene which encodes a quinone oxidoreductase-like protein-2, which is involved in resistance against *Phytophthora infestans*, the causal agent of potato late blight [49]. Furthermore, *Cp4.1LG13g07410* gene encoding a BHLH transcription factor previously implicated in conferring resistance against *Phytophthora sojae* in soybean and *Phytophthora parasitica* in tobacco [50,51] was found 0.16 Mb downstream of SNP C002686. Three other genes (*Cp4.1LG13g08020*, *Cp4.1LG13g08190*, and *Cp4.1LG13g09000*) encoding a RING/U-box superfamily protein, Methyl esterase-11 and L-aspartate oxidase, respectively, were found upstream of SNP C002686. RING/U-box proteins activate plant defense mechanisms in plants and play an important role in defense against fungal pathogens, including *Cladosporium fulvum*, the causal agent of leaf mold in tomato [52]. On the other hand, *Methyl esterase-11* and *L-aspartate oxidase* genes inhibit the development of fungal diseases by enhancing pectin esterification and through NAD^+^ regulation, respectively [53,54]. SNP C009351 lies within a dirigent protein gene *Cp4.1LG13g09560* which confers resistance to powdery mildew in Cucurbitaceae family [55]. SNP marker C010730 was located within *Cp4.1LG13g07250*, a gene encoding a Eukaryotic translation initiation factor 3 subunit F involved in resistance against papaya ringspot virus in papaya [56]. SNP marker C030107 is found within *Cp4.1LG13g10990*, a gene encoding E3 ubiquitin-protein ligase (SDIR1) that modulates plant innate immunity, broad spectrum disease resistance and abiotic stress responses [57]. 

Previous studies involving *P. capsici* in squash showed that the pathogen stimulates the production of reactive oxygen species resulting in cell death, while hyphae colonization occludes vascular bundles inhibiting water and nutrient mobility [13]. The functional mechanisms of the candidate resistance genes within *QtlPC-C13* suggest that resistance in #181761-36P maybe conferred through anti-oxidative defense and structural reinforcement of vascular bundles that inhibit pathogen penetration. Similar resistance mechanisms have been reported for other soil borne pathogens in soybean [58] and pepper Dunn and Smart [59]. 

## 5. Conclusions

A major QTL (*QtlPC-C13)* associated with resistance to Phytophthora crown rot in #181761-36P was mapped on chromosome 13 of the *C. pepo* genome. SNP marker C002686 was significantly linked to Phytophthora crown rot resistance and is a potential target for MAS in squash breeding programs. *QtlPC-C13* in #181761-36P can be used to complement resistance to Phytophthora crown rot in breeding line Pc-NY21 [10] to confer durable resistance. Several candidate disease resistance genes, including those involved in resistance against Phytophthora syndromes in other crops were identified within *QtlPC-C13*. Functional analysis of these genes is needed to elucidate the molecular mechanisms underlying resistance to Phytophthora crown rot in *C. pepo.*

## Figures and Tables

**Table 1:** Linkage map (length = 2068.96 cM) for the F_2_ population derived from a cross between #181761-36P (resistant) and Table Queen (susceptible). The linkage map had 21 linkage groups and consisted of 244 SNP markers with a marker density of 8.1 SNP/ cM.

Chromosome	Number of Genotyped SNPs	Number of Mapped SNPs	Length of Linkage Group (cM)	Average Number of SNPs per cM
Cp4.1LG00	3	2	0.37	0.2
Cp4.1LG01	45	19	243.89	12.8
Cp4.1LG02	30	11	89.73	8.2
Cp4.1LG03	36	22	237.40	10.8
Cp4.1LG04	24	12	107.93	9.0
Cp4.1LG05	32	17	111.61	6.6
Cp4.1LG06	25	13	84.08	6.5
Cp4.1LG07	18	12	84.52	7.0
Cp4.1LG08	31	16	121.01	7.6
Cp4.1LG09	28	8	120.54	15.1
Cp4.1LG10	29	19	109.62	5.8
Cp4.1LG11	32	7	90.74	13.0
Cp4.1LG12	36	17	133.56	7.9
Cp4.1LG13	25	14	98.99	7.1
Cp4.1LG14	14	6	87.81	14.6
Cp4.1LG15	19	12	68.61	5.7
Cp4.1LG16	24	6	55.86	9.3
Cp4.1LG17	6	2	0.75	0.4
Cp4.1LG18	23	9	55.21	6.1
Cp4.1LG19	24	10	102.36	10.2
Cp4.1LG20	19	10	64.34	6.4
Total	523	244	2068.96	Mean = 8.1

**Table 2:** Linkage group positions (cM) of the QTL associated with resistance to Phytophthora crown rot on chromosome 13 and the corresponding peak SNP positions in the #181761-36P × Table Queen F_2:3_ squash population.

Screen	Position (cM)	R^2^	LOD	Peak SNP	Additive Effect	Dominance Effect
Experiment 1	89.3	17.92	3.66	C002686	4.14	1.75
Experiment 2	97.9	18.47	3.14	C002686	3.14	2.84
Experiment 3	90.5	21.47	4.04	C002686	3.81	2.78
Joint Analysis	89.7	20.79	5.92	C002686	3.88	2.41

## Data Availability

Not applicable.

## Supplementary Materials

The following are available online at https://www.mdpi.com/article/10.3390/plants10102115/s1, Table S1: Chromosomal locations of six hundred and five SNP markers targeted for genotyping in the F_2_ population between #181761-36P (resistant) and Table Queen cultivar (susceptible). Table S2: Descriptive data for the five SNPs within the QTL interval (*QtlPC-C13*) that were genotyped in the F_2_ population between #181761-36P (resistant) and Table Queen cultivar (susceptible). Table S3: Genome (*Cucurbita pepo* v4.1) coverage statistics for the linkage map constructed for the F_2_ population between #181761-36P (resistant) and Table Queen cultivar (susceptible). Table S4: Twenty-three genes within the QTL interval (*QtlPC-C13*). Candidate disease resistance genes are highlighted in green. Figure S1: A linkage map comprising of 21 linkage groups and 244 single nucleotide polymorphism markers for the F_2_ population between #181761-36P (resistant) and Table Queen cultivar (susceptible).
