# Supplementary material for: A Novel QTL for Resistance to Phytophthora Crown Rot in Squash
Source: Plants (Basel). 2021 Oct 6;10(10):2115. doi: 10.3390/plants10102115 (PMC8537320; doi:10.3390/plants10102115)
Supplement: Supplementary file 1 [file plants-10-02115-s001.zip › Figure S1_Linkage Map.pdf]

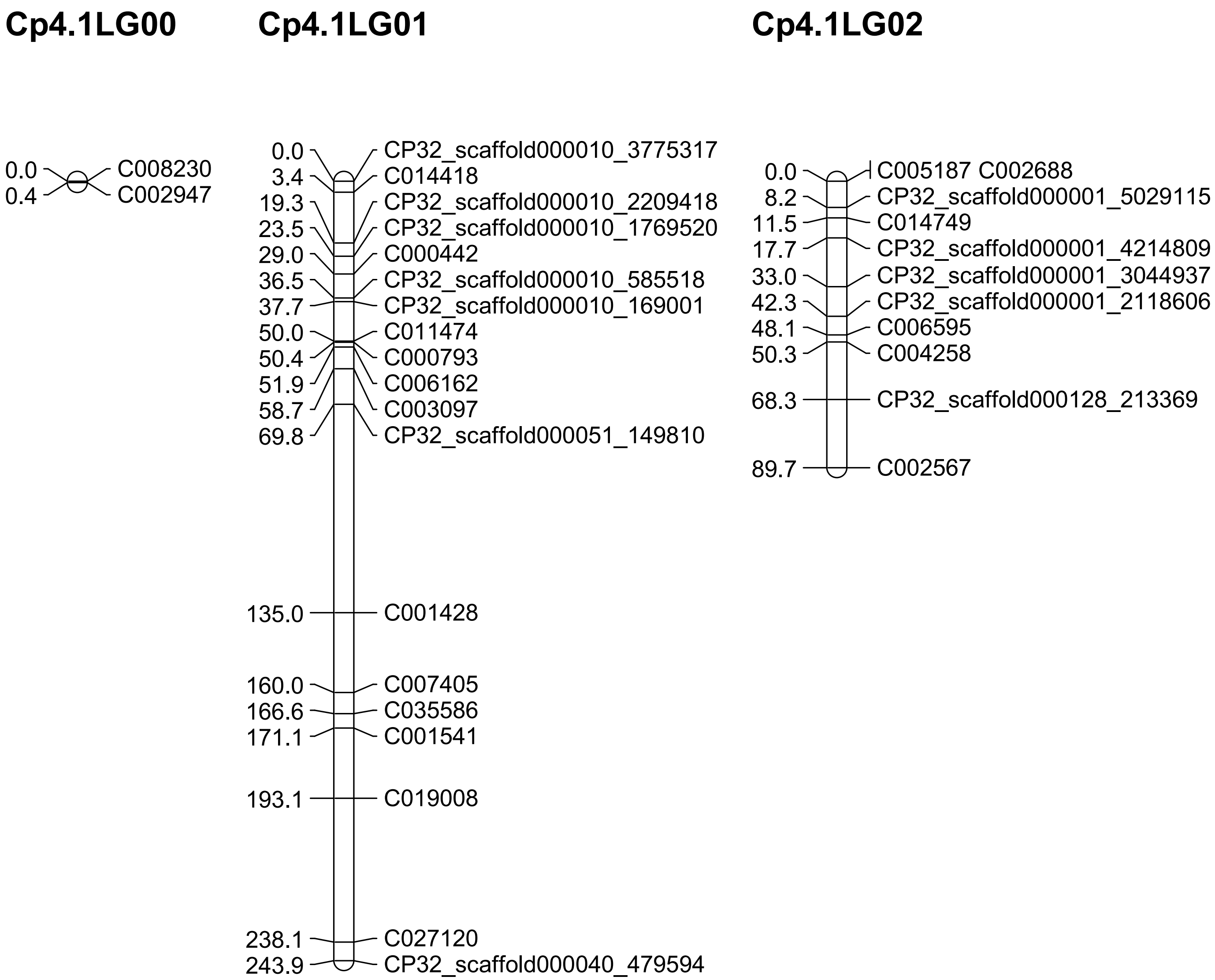
Cp4.1LG00
0.0 C008230
0.4 C002947
Cp4.1LG01
0.0 CP32_scaffold000010_3775317
3.4 C014418
19.3 CP32_scaffold000010_2209418
23.5 CP32_scaffold000010_1769520
29.0 C000442
36.5 CP32_scaffold000010_585518
37.7 CP32_scaffold000010_169001
50.0 C011474
50.4 C000793
51.9 C006162
58.7 C003097
69.8 CP32_scaffold000051_149810
135.0 C001428
160.0 C007405
166.6 C035586
171.1 C001541
193.1 C019008
238.1 C027120
243.9 CP32_scaffold000040_479594
Cp4.1LG02
0.0 C005187 C002688
8.2 CP32_scaffold000001_5029115
11.5 C014749
17.7 CP32_scaffold000001_4214809
33.0 CP32_scaffold000001_3044937
42.3 CP32_scaffold000001_2118606
48.1 C006595
50.3 C004258
68.3 CP32_scaffold000128_213369
89.7 C002567

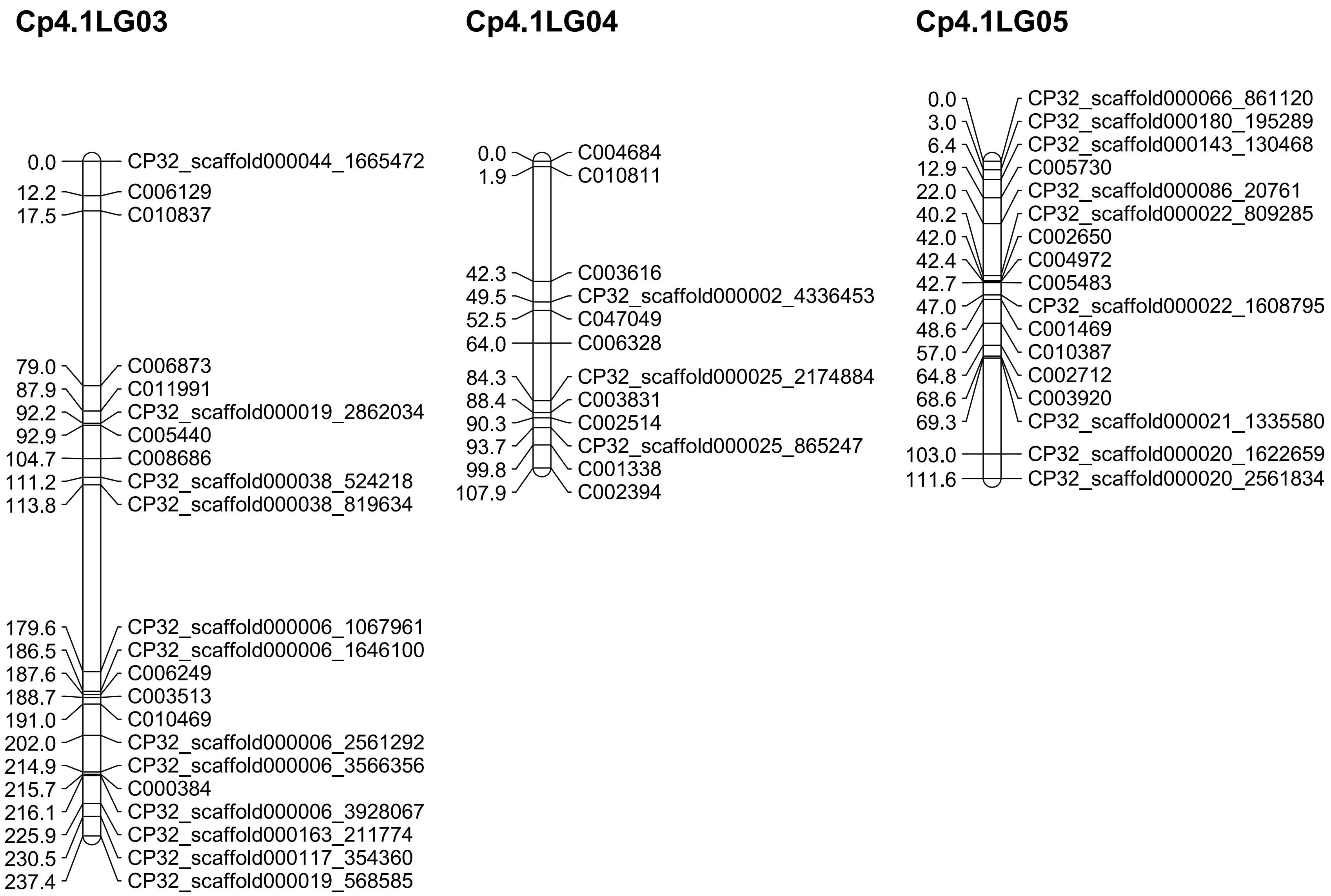
Cp4.1LG03
0.0 CP32_scaffold000044_1665472
12.2 C006129
17.5 C010837
79.0 C006873
87.9 C011991
92.2 CP32_scaffold000019_2862034
92.9 C005440
104.7 C008686
111.2 CP32_scaffold000038_524218
113.8 CP32_scaffold000038_819634
179.6 CP32_scaffold000006_1067961
186.5 CP32_scaffold000006_1646100
187.6 C006249
188.7 C003513
191.0 C010469
202.0 CP32_scaffold000006_2561292
214.9 CP32_scaffold000006_3566356
215.7 C000384
216.1 CP32_scaffold000006_3928067
225.9 CP32_scaffold000163_211774
230.5 CP32_scaffold000117_354360
237.4 CP32_scaffold000019_568585
Cp4.1LG04
0.0 C004684
1.9 C010811
42.3 C003616
49.5 CP32_scaffold000002_4336453
52.5 C047049
64.0 C006328
84.3 CP32_scaffold000025_2174884
88.4 C003831
90.3 C002514
93.7 CP32_scaffold000025_865247
99.8 C001338
107.9 C002394
Cp4.1LG05
0.0 CP32_scaffold000066_861120
3.0 CP32_scaffold000180_195289
6.4 CP32_scaffold000143_130468
12.9 C005730
22.0 CP32_scaffold000086_20761
40.2 CP32_scaffold000022_809285
42.0 C002650
42.4 C004972
42.7 C005483
47.0 CP32_scaffold000022_1608795
48.6 C001469
57.0 C010387
64.8 C002712
68.6 C003920
69.3 CP32_scaffold000021_1335580
103.0 CP32_scaffold000020_1622659
111.6 CP32_scaffold000020_2561834

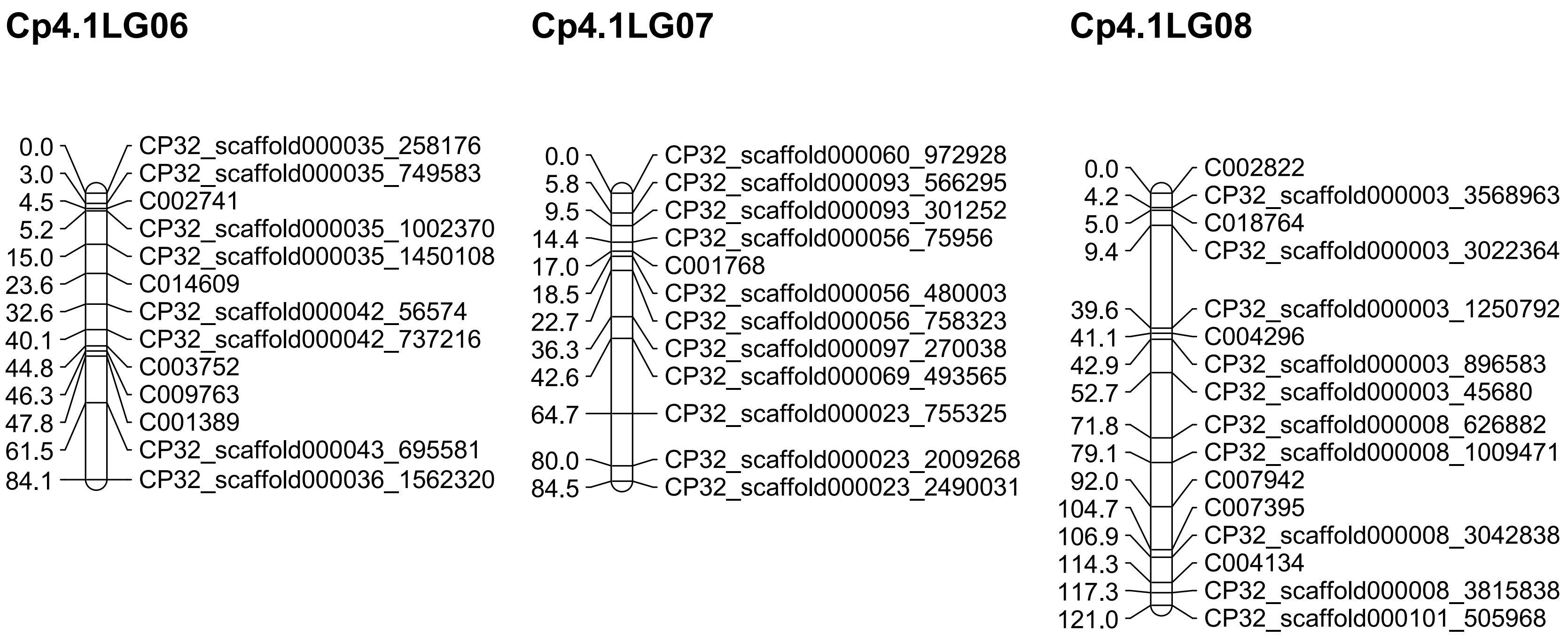
Cp4.1LG06
0.0 CP32_scaffold000035_258176
3.0 CP32_scaffold000035_749583
4.5 C002741
5.2 CP32_scaffold000035_1002370
15.0 CP32_scaffold000035_1450108
23.6 C014609
32.6 CP32_scaffold000042_56574
40.1 CP32_scaffold000042_737216
44.8 C003752
46.3 C009763
47.8 C001389
61.5 CP32_scaffold000043_695581
84.1 CP32_scaffold000036_1562320
Cp4.1LG07
0.0 CP32_scaffold000060_972928
5.8 CP32_scaffold000093_566295
9.5 CP32_scaffold000093_301252
14.4 CP32_scaffold000056_75956
17.0 C001768
18.5 CP32_scaffold000056_480003
22.7 CP32_scaffold000056_758323
36.3 CP32_scaffold000097_270038
42.6 CP32_scaffold000069_493565
64.7 CP32_scaffold000023_755325
80.0 CP32_scaffold000023_2009268
84.5 CP32_scaffold000023_2490031
Cp4.1LG08
0.0 C002822
4.2 CP32_scaffold000003_3568963
5.0 C018764
9.4 CP32_scaffold000003_3022364
39.6 CP32_scaffold000003_1250792
41.1 C004296
42.9 CP32_scaffold000003_896583
52.7 CP32_scaffold000003_45680
71.8 CP32_scaffold000008_626882
79.1 CP32_scaffold000008_1009471
92.0 C007942
104.7 C007395
106.9 CP32_scaffold000008_3042838
114.3 C004134
117.3 CP32_scaffold000008_3815838
121.0 CP32_scaffold000101_505968

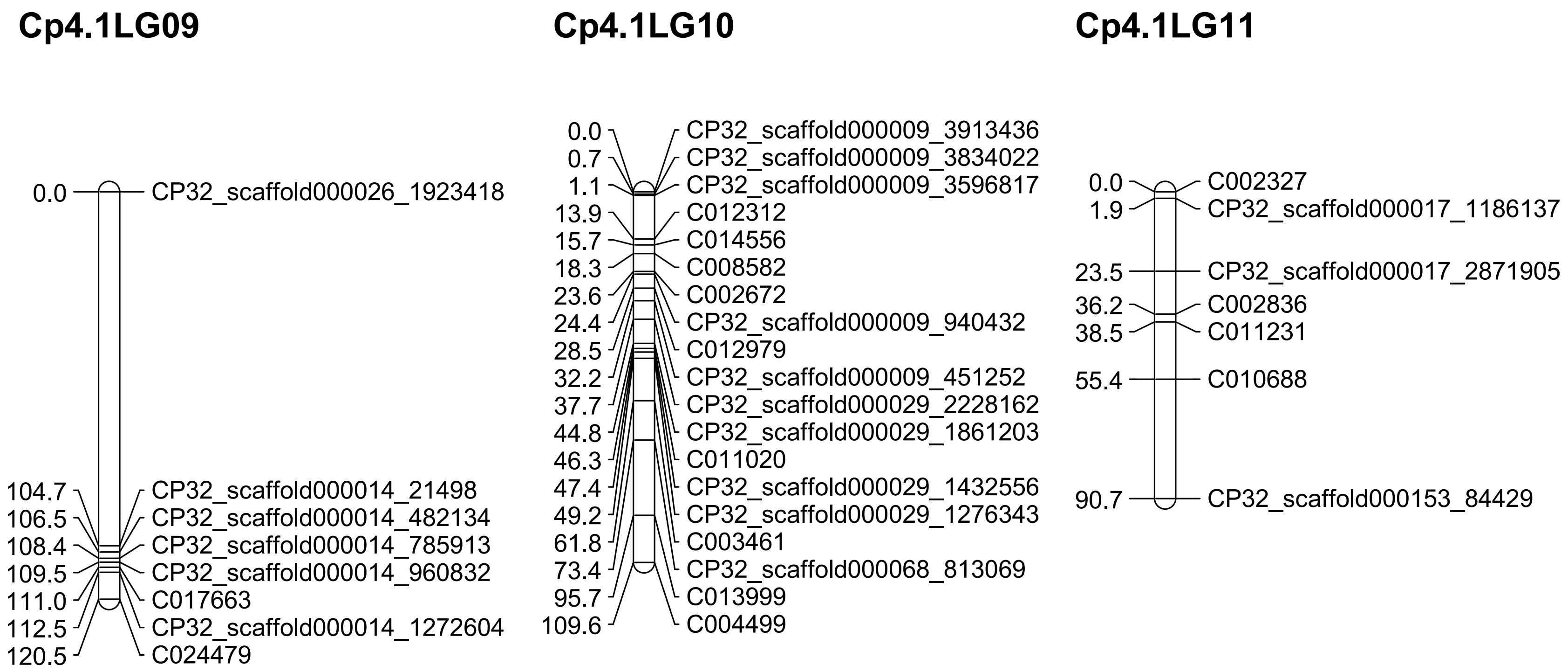
Cp4.1LG09
0.0 CP32_scaffold000026_1923418
104.7 CP32_scaffold000014_21498
106.5 CP32_scaffold000014_482134
108.4 CP32_scaffold000014_785913
109.5 CP32_scaffold000014_960832
111.0 C017663
112.5 CP32_scaffold000014_1272604
120.5 C024479
Cp4.1LG10
0.0 CP32_scaffold000009_3913436
0.7 CP32_scaffold000009_3834022
1.1 CP32_scaffold000009_3596817
13.9 C012312
15.7 C014556
18.3 C008582
23.6 C002672
24.4 CP32_scaffold000009_940432
28.5 C012979
32.2 CP32_scaffold000009_451252
37.7 CP32_scaffold000029_2228162
44.8 CP32_scaffold000029_1861203
46.3 C011020
47.4 CP32_scaffold000029_1432556
49.2 CP32_scaffold000029_1276343
61.8 C003461
73.4 CP32_scaffold000068_813069
95.7 C013999
109.6 C004499
Cp4.1LG11
0.0 C002327
1.9 CP32_scaffold000017_1186137
23.5 CP32_scaffold000017_2871905
36.2 C002836
38.5 C011231
55.4 C010688
90.7 CP32_scaffold000153_84429

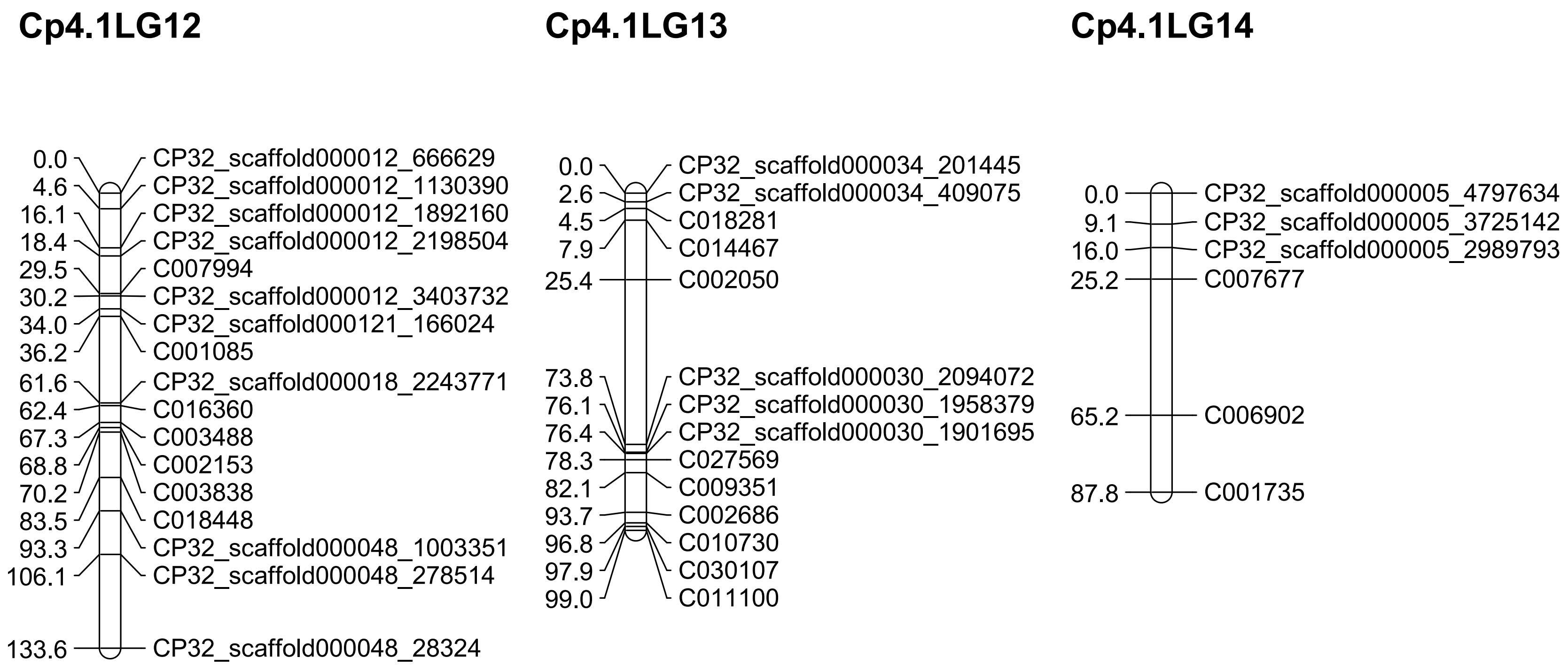
Cp4.1LG12
0.0 CP32_scaffold000012_666629
4.6 CP32_scaffold000012_1130390
16.1 CP32_scaffold000012_1892160
18.4 CP32_scaffold000012_2198504
29.5 C007994
30.2 CP32_scaffold000012_3403732
34.0 CP32_scaffold000121_166024
36.2 C001085
61.6 CP32_scaffold000018_2243771
62.4 C016360
67.3 C003488
68.8 C002153
70.2 C003838
83.5 C018448
93.3 CP32_scaffold000048_1003351
106.1 CP32_scaffold000048_278514
133.6 CP32_scaffold000048_28324
Cp4.1LG13
0.0 CP32_scaffold000034_201445
2.6 CP32_scaffold000034_409075
4.5 C018281
7.9 C014467
25.4 C002050
73.8 CP32_scaffold000030_2094072
76.1 CP32_scaffold000030_1958379
76.4 CP32_scaffold000030_1901695
78.3 C027569
82.1 C009351
93.7 C002686
96.8 C010730
97.9 C030107
99.0 C011100
Cp4.1LG14
0.0 CP32_scaffold000005_4797634
9.1 CP32_scaffold000005_3725142
16.0 CP32_scaffold000005_2989793
25.2 C007677
65.2 C006902
87.8 C001735

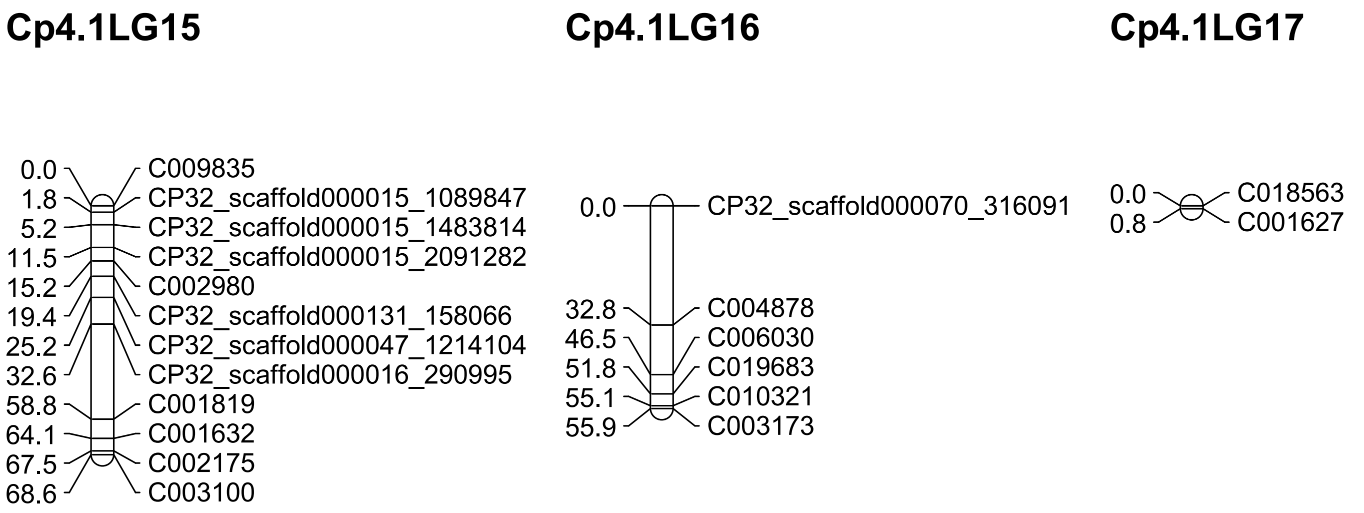
Cp4.1LG15
0.0 C009835
1.8 CP32_scaffold000015_1089847
5.2 CP32_scaffold000015_1483814
11.5 CP32_scaffold000015_2091282
15.2 C002980
19.4 CP32_scaffold000131_158066
25.2 CP32_scaffold000047_1214104
32.6 CP32_scaffold000016_290995
58.8 C001819
64.1 C001632
67.5 C002175
68.6 C003100
Cp4.1LG16
0.0 CP32_scaffold000070_316091
32.8 C004878
46.5 C006030
51.8 C019683
55.1 C010321
55.9 C003173
Cp4.1LG17
0.0 C018563
0.8 C001627

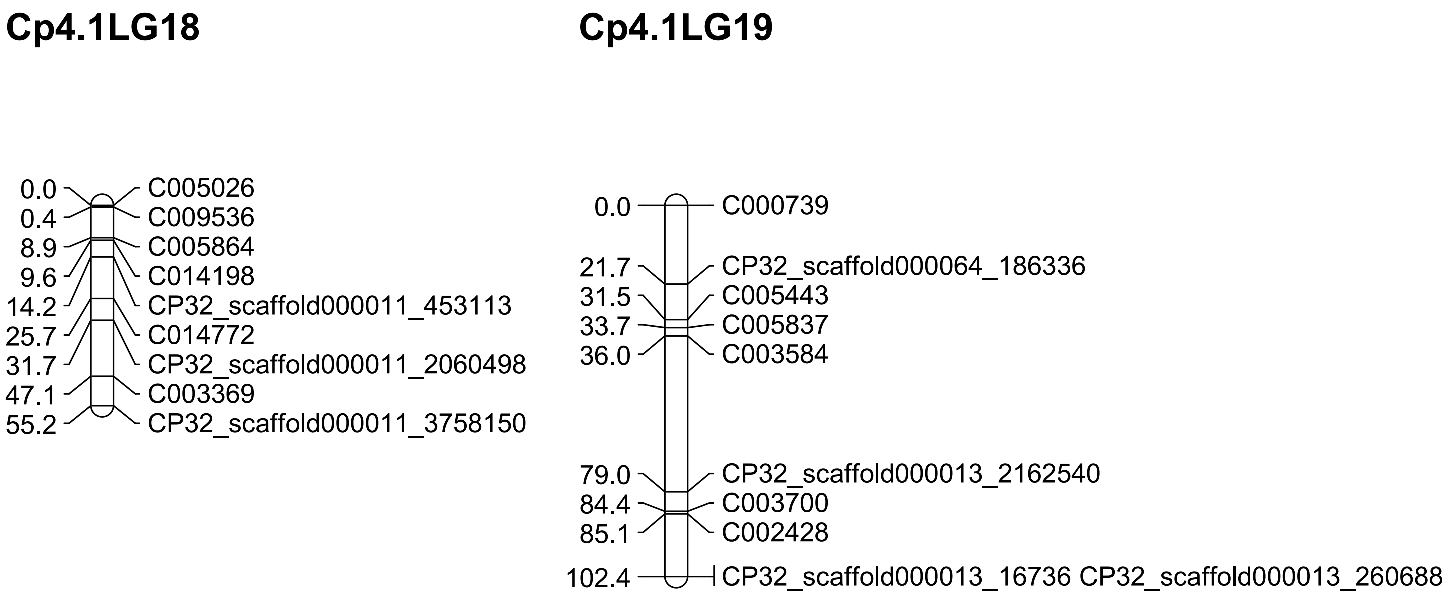
Cp4.1LG18
0.0 C005026
0.4 C009536
8.9 C005864
9.6 C014198
14.2 CP32_scaffold000011_453113
25.7 C014772
31.7 CP32_scaffold000011_2060498
47.1 C003369
55.2 CP32_scaffold000011_3758150
Cp4.1LG19
0.0 C000739
21.7 CP32_scaffold000064_186336
31.5 C005443
33.7 C005837
36.0 C003584
79.0 CP32_scaffold000013_2162540
84.4 C003700
85.1 C002428
102.4 CP32_scaffold000013_16736 CP32_scaffold000013_260688

Cp4.1LG20

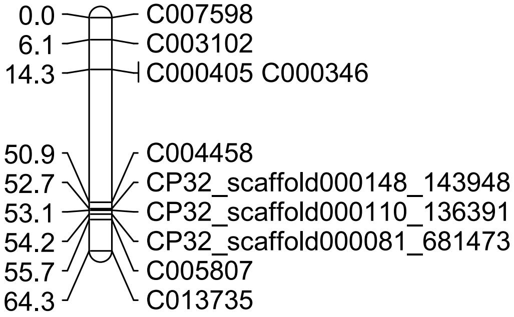

0.0
6.1
14.3
50.9
52.7
53.1
54.2
55.7
64.3
C007598
C003102
C000405 C000346
C004458
CP32_scaffold000148_143948
CP32_scaffold000110_136391
CP32_scaffold000081_681473
C005807
C013735
